# Psychometric properties of the Portuguese version of the Personality Assessment Inventory: normative data and reliability

**DOI:** 10.3389/fpsyg.2024.1359793

**Published:** 2024-05-30

**Authors:** Mauro Paulino, Mariana Moniz, Octávio Moura, Daniel Rijo, Leslie Morey, Mário R. Simões

**Affiliations:** ^1^Center for Research in Neuropsychology and Cognitive and Behavioral Intervention (CINEICC), University of Coimbra, Coimbra, Portugal; ^2^Psychological Assessment and Psychometrics Laboratory (PsyAssessmentLab), University of Coimbra, Coimbra, Portugal; ^3^Universidade Europeia, Faculty of Health Sciences, Lisbon, Portugal; ^4^Institute of Clinical and Forensic Psychology (MIND), Lisbon, Portugal; ^5^Faculty of Psychology and Educational Sciences, University of Coimbra, Coimbra, Portugal; ^6^College of Arts & Sciences, Texas A&M University Central Texas, Killeen, TX, United States

**Keywords:** Personality Assessment Inventory (PAI), Portuguese validation, psychometric properties, normative data, reliability

## Abstract

**Introduction:**

Originally published in the United States of America in 1991, the Personality Assessment Inventory (PAI) has been translated and adapted to a growing number of countries, but Portugal had yet to study its adequacy to the Portuguese population.

**Methods:**

The current study aimed to investigate the Portuguese normative data, the predictive effect of sociodemographic variables on the PAI scores, and the reliability of the Portuguese version of the PAI. Additionally, results were compared with other international versions of the PAI. The sample was comprised of 900 participants (age: *M* = 43.13, *SD* = 14.28, range = 18–75), recruited from various regions of Portugal.

**Results:**

Findings showed that the Portuguese sample scored higher than the U.S. and other international versions of the PAI in most scales. Sociodemographic variables (e.g., gender, age, and educational level) were significant predictors on PAI scores. The internal consistency of the Portuguese sample revealed lower values on the validity scales, but adequate on the clinical, treatment, and interpersonal scales. Overall, the Portuguese PAI revealed adequate psychometric properties, with normative results often superior to other international versions of the inventory.

**Discussion:**

It is a crucial step into the Portuguese adaptation and validation of this instrument, a measure with considerable potential in clinical, forensic, and research contexts. This adaptation may lead to the growth and development of the psychological assessment field in Portugal, and the opportunity to develop future cross-cultural studies with other international versions of the PAI.

## Introduction

The Personality Assessment Inventory (PAI) is a multiscale self-report measure of psychopathology and personality developed in 1991 by Leslie Morey. This inventory aims to provide information on critical variables for diagnostic and clinical decision-making purposes. The PAI includes 344 items, organized into 22 non-overlapping scales, resulting from a careful theoretical and empiric review, and items within each scale were selected based on their substantial associations with relevant constructs (e.g., response validity, clinical syndromes, interpersonal style, treatment complications, and environment characteristics) while considering their minimal associations with items of different constructs. Thus, in contrast to other self-report personality inventories (e.g., the Minnesota Multiphasic Personality Inventories or the Millon Clinical Multiaxial Inventories), none of the PAI scales overlapped with the remaining ones (Morey and McCredie, 2019). The PAI includes four validity scales, 11 clinical scales, five treatment consideration scales, and two interpersonal scales (Morey, 1991) (see Table 1).

**Table 1:** PAI structure.

	Scales	Subscales
Validity scales	Inconsistency (ICN)	
	Infrequency (INF)	
	Negative impression (NIM)	
	Positive impression (PIM)	
Clinical scales	Somatic Complaints (SOM)	Conversion (SOM-C)
		Health concerns (SOM-H)
		Somatization (SOM-S)
	Anxiety (ANX)	Cognitive (ANX-C)
		Affective (ANX-A)
		Physiological (ANX-P)
	Anxiety-related disorders (ARD)	Obsessive-compulsive (ARD-O)
		Phobias (ARD-P)
		Traumatic stress (ARD-T)
	Depression (DEP)	Cognitive (DEP-C)
		Affective (DEP-A)
		Physiological (DEP-P)
	Mania (MAN)	Activity level (MAN-A)
		Grandiosity (MAN-G)
		Irritability (MAN-I)
	Paranoia (PAR)	Hypervigilance (PAR-H)
		Persecution (PAR-P)
		Resentment (PAR-R)
	Schizophrenia (SCZ)	Psychotic experience (SCZ-P)
		Social detachment (SCZ-S)
		Thought disorder (SCZ-T)
	Borderline features (BOR)	Affective instability (BOR-A)
		Identity problems (BOR-I)
		Negative relationships (BOR-N)
		Self-harm (BOR-S)
	Antisocial features (ANT)	Antisocial behaviors (ANT-A)
		Egocentricity (ANT-E)
		Stimulus-seeking (ANT-S)
	Alcohol problems (ALC)	
	Drug problems (DRG)	
Treatment scales	Aggression (AGG)	Aggressive attitude (AGG-A)
		Verbal aggression (AGG-V)
		Physical aggression (AGG-P)
	Suicidal Ideation (SUI)	
	Stress (STR)	
	Nonsupport (NON)	
	Treatment Rejection (RXR)	
Interpersonal scales	Dominance (DOM) Warmth (WRM)	

The four validity scales – Inconsistency (ICN), Infrequency (INF), Negative Impression (NIM), and Positive Impression (PIM) – were developed to measure systematic or random profile distortions (Morey and McCredie, 2020). The clinical scales measure the most relevant clinical domains in the field of mental illness, which gathered the most focus in contemporary diagnostic practices (Morey, 1991). These include the Somatic Complaints (SOM), Anxiety (ANX), Anxiety-Related Disorders (ARD), Depression (DEP), Mania (MAN), Paranoia (PAR), Schizophrenia (SCZ), Borderline Features (BOR), Antisocial Features (ANT), Alcohol Problems (ALC), and Drug Problems (DRG) scales (Morey, 1991). These scales (excepting the ALC and DRG scales) are organized into several subscales, each of them related to a particular clinical syndrome. The treatment consideration scales aim to assist professionals in decision-making process by providing information regarding the risk the examinees present, regarding themselves and others. These scales include the Aggression (AGG), Suicidal Ideation (SUI), Stress (STR), Nonsupport (NON), and Treatment Rejection (RXR) scales. Additionally, the AGG scale incorporates some subscales, covering different components of aggression (Morey, 1991). Lastly, the interpersonal scales – Dominance (DOM) and Warmth (WRM) – assess interpersonal behaviors, making them an important tool in the therapeutic process (Kiesler, 1996). The interpersonal scales also provide information concerning variations in normal personality and are sensitive to the presence of possible mental pathologies, following the belief that the most important expressions of personality occur in events that involve more than one person (Pincus, 2005).

In addition to the scales, several supplemental indexes are presented in the professional manual, namely the Malingering Index (MAL), the Rogers Discriminant Function (RDF), the Defensiveness Index (DEF), the Cashel Discriminant Function (CDF), the Suicide Potential Index (SPI), the Violence Potential Index, and the Treatment Process Index (TPI) (Morey, 1991). Even though the RDF and CDF have lost support in past decades as reliable indicators of response style (e.g., Hawes and Boccaccini, 2009), other indicators, such as the Multi-Feign Index (MFI; Gaines et al., 2013) and the Cognitive Bias Scale (CBS; Gaasedelen et al., 2019), have steadily been gaining support as relevant supplemental indexes (e.g., Ingram et al., 2024).

The PAI can be completed individually or in group resorting to an Answer Sheet or an online administration and scoring via PARiConnect. Minimal differences have been found between these administration methods, so results should be considered interchangeable if the appropriate guidelines are followed (Finger and Ones, 1999; Morey, 2007; American Psychological Association, 2020). For example, a recent study compared Minnesota Multiphasic Personality Inventory-2-Restructured Form (MMPI-2-RF) scores of police candidates who completed the test in an in-person condition with the scores of candidates who completed it remotely. The results revealed that the normative data and psychometric properties were equivalent regardless of whether the test was administered in-person or remotely, with group differences no greater than two T-score points per scale (Menton et al., 2022). Overall, a growing body of research has supported the use of telehealth alternatives to in-person assessment procedures, with minimal to no loss of scale effectiveness (e.g., Corey and Ben-Porath, 2020; Agarwal et al., 2023).

The PAI has shown great utility in different settings, such as research and training (e.g., Schroeder et al., 2016; Stedman et al., 2017), and in forensic (e.g., Ackerman et al., 2021) and neuropsychology contexts (e.g., Martin et al., 2015). For instance, in personality assessment training and practice settings, it is one of the most used instruments, providing clinical practice and higher self-report competency to trainees (e.g., Mihura et al., 2017; Ingram et al., 2022), and, in forensic cases, it appears to be a promising measure of malingering and a good predictor of inmate violence, misconduct, and recidivism (e.g., Reidy et al., 2016).

PAI reliability has been heavily studied and the professional manual (Morey, 1991) presented adequate internal consistency (full scale Cronbach alpha mean of 0.81 in a census sample, 0.82 in a college sample, and 0.86 in a clinical sample) and test–retest reliability (average *r* = 0.82 across the 22 scales). The lower Cronbach alphas belonged to two validity scales, particularly the Inconsistency (e.g., 0.23 in the clinical sample) and Infrequency (e.g., 0.40 in the clinical sample). Boyle and Lennon (1994) also found adequate alpha coefficients (median = 0.83) in a sample of 211 subjects. Similar findings were also reported by other international adaptations of the inventory. For example, Burneo-Garcés et al. (2020) found alpha coefficients ranging from 0.49 (PIM) to 0.89 (DRG) for the Spanish adaptation of the PAI, Groves and Engel (2007) reported alphas ranging from 0.26 (INF) to 0.91 (SOM) for the German version of the inventory, and Bach-Nguyen and Morey (2018) found alphas ranging from 0.43 (ICN) to 0.94 (SUI) for the Vietnamese version. In general, the lowest alphas belonged to the validity scales, particularly ICN and INF which were designed as measures of random error. Morey (1991) argued that because these two validity scales do not measure substantive theoretical constructs, but instead are comprised of indicators of random error variance, it would be expected that the items could be uncorrelated.

The influence of sociodemographic variables on the PAI scales (e.g., age, gender, and education) were observed in the original study of Morey (1991). The author found that age exhibited some influence on PAI scores, where younger adults scored significantly higher in PAR, BOR, and ANT. Interestingly, SOM scores appeared to be stable across age ranges, suggesting that this scale does not reflect the types of physical disorders experienced by elderly people. Differences in gender were small, except on the ANT and ALC scales, where men scored significantly more than women. Finally, education appeared to present consistent effect across scales; therefore, people with lower years of education scored, generally, above the mean. Despite these findings, no other studies that focused on the impact of sociodemographic variables on PAI scores in a normative sample were found.

The PAI has been translated and adapted to a growing number of countries, including China, Germany, Spain, Greece, Italy, Mexico, Argentina, Vietnam, Iran, South-Korea, and Canada. Of these international versions of the PAI, South-Korea presented the highest standardization sample (*n* = 2,212), while Iran resorted to the smallest one (*n* = 462).

Overall, the translation and adaptation of psychological tests allows the evaluation of people from different countries by well-established measures in their own language and culture. Furthermore, test translations and adaptations, if done according to recognized guidelines and procedures (e.g., International Test Commission (ITC), 2017), facilitate the development of cross-cultural studies, reducing costs and saving time in developing new tests, while providing increasingly robust data and support to the validity and reliability of the adapted measures (Iliescu, 2017; Hernández et al., 2020). To ensure an adequate test adaptation and validation, researchers should provide evidence supporting the norms, reliability, and validity of the adapted version, thereby guaranteeing that the obtained norms are adequate for interpreting test scores (Hernández et al., 2020). The establishment of norms is of particular importance, given that they are statistical data that summarize the distribution of test scores for one or more groups, with the aim of representing a given reference to the population (American Educational Research Association et al., 2014).

The current study aims to investigate the normative data, the predictive effect of sociodemographic variables on the scales scores, and the reliability of the Portuguese version of the PAI. We also compared the Portuguese version with other international versions.

## Method

### Participants

Data collection was carried out from April 2021 to March 2022. The initial sample was comprised of 2,362 participants, recruited online from various regions of Portugal (i.e., North, Center, Lisbon, Alentejo, Algarve, Madeira, and Azores). In accordance with the PAI Manual (Morey, 1991), participants were dropped out if the scores of the validity scales were above the cut-off scores. Sociodemographic variables were collected, and participants were selected based on specific stratified criteria (i.e., gender, age, education, and region) close to the Portuguese census (PORDATA, 2021).^1^ Participants were also excluded if they answered *Yes* to either of these two questions: “*In the last year, did you receive Neurological or Psychiatric treatment?”* or “*In the last year, did you have any problems with the law?”*

From the original 2,362 participants, 1,297 were eliminated from the final sample due to stratification criteria, and 165 were eliminated due to invalid profiles. Consequently, a total of 900 individuals were included in the final sample (Portuguese standardization sample of the PAI), 381 (42.30%) of which were male and 519 (57.70%) were female. Descriptive statistics for the sociodemographic characteristics of the final sample are reported in Table 2 and cumulative elevation frequencies related to various *T* score cutoffs across gender are presented in Table 3.

**Table 2:** Sociodemographic characteristics of the PAI Portuguese standardization sample.

Variable	*n*	%
Age (years)		
18–24	108	12.00
25–29	88	9.80
30–39	173	19.20
40–49	217	24.10
50–59	174	19.30
60–74	140	15.60
Gender		
Male	381	42.30
Female	519	57.70
Education level		
Primary education	22	2.40
Middle school	68	7.60
High school	307	34.10
Tertiary education	503	55.90
Region		
North	237	26.30
Center	192	21.30
Lisbon	301	33.40
Alentejo	68	7.60
Algarve	46	5.10
Madeira	31	3.40
Azores	25	2.80

**Table 3:** PAI normative data for the Portuguese sample by gender (raw scores) and frequency of scale elevations (T-scores).

Scales	Gender	*M*	*SD*	*t* (898)	*p*	*Cohen’s d*	Kurtosis	Skew	Cumulative % of administrations ≥ T-score						
									60	70	80	90	100	110	120
Validity scales															
ICN	Female	5.19	2.28	−2.93	0.003	0.20	−0.06	0.38	13.7	2.1	0.0	0.0	0.0	0.0	0.0
	Male	5.66	2.50				−0.77	0.17	22.6	2.4	0.0	0.0	0.0	0.0	0.0
INF	Female	3.24	2.08	−2.80	0.005	0.19	−0.75	0.25	13.8	2.1	0.0	0.0	0.0	0.0	0.0
	Male	3.63	2.03				−0.77	0.19	16.0	2.9	0.0	0.0	0.0	0.0	0.0
NIM	Female	2.08	2.54	0.91	0.359	0.06	3.14	1.74	7.9	5.0	1.4	1.0	0.0	0.0	0.0
	Male	1.93	2.44				3.64	1.85	5.8	5.2	0.8	0.8	0.0	0.0	0.0
PIM	Female	15.60	3.93	−0.68	0.498	0.05	0.06	−0.60	16.0	0.0	0.0	0.0	0.0	0.0	0.0
	Male	15.78	3.98				> 0.01	−0.62	17.6	0.0	0.0	0.0	0.0	0.0	0.0
Clinical scales															
SOM	Female	14.10	9.85	3.11	0.002	0.21	0.45	0.97	10.4	5.6	1.6	0.0	0.0	0.0	0.0
	Male	12.20	8.44				1.90	1.41	6.6	4.5	0.3	0.0	0.0	0.0	0.0
ANX	Female	25.01	12.54	4.96	<0.001	0.33	0.38	0.84	14.3	4.6	1.5	0.0	0.0	0.0	0.0
	Male	21.06	11.25				0.92	0.99	8.9	2.9	0.3	0.0	0.0	0.0	0.0
ARD	Female	23.09	8.71	4.42	<0.001	0.32	1.06	0.89	12.7	3.7	1.0	0.2	0.0	0.0	0.0
	Male	20.26	9.21				1.40	1.09	9.7	2.9	0.8	0.0	0.0	0.0	0.0
DEP	Female	20.24	10.66	1.94	0.054	0.13	0.79	0.99	10.6	5.2	1.4	0.0	0.0	0.0	0.0
	Male	18.85	10.72				1.40	1.11	8.7	3.9	1.6	0.0	0.0	0.0	0.0
MAN	Female	20.59	8.39	−2.62	0.010	0.18	0.52	0.64	11.2	2.3	0.8	0.0	0.0	0.0	0.0
	Male	22.11	8.93				0.55	0.58	11.3	4.7	1.0	0.0	0.0	0.0	0.0
PAR	Female	23.82	9.45	−2.50	0.013	0.17	0.14	0.50	12.9	2.8	0.2	0.1	0.0	0.0	0.0
	Male	25.45	9.95				0.84	0.83	13.6	4.5	1.0	0.3	0.0	0.0	0.0
SCZ	Female	15.98	8.53	0.07	0.945	0.00	0.54	0.84	13.5	3.3	1.3	0.0	0.0	0.0	0.0
	Male	15.94	7.97				1.76	1.09	11.5	2.9	0.3	0.6	0.0	0.0	0.0
BOR	Female	23.34	10.22	0.85	0.399	0.06	0.06	0.60	13.1	3.7	0.6	0.0	0.0	0.0	0.0
	Male	22.74	10.52				0.25	0.81	12.6	4.7	0.3	0.0	0.0	0.0	0.0
ANT	Female	11.11	5.83	−9.17	<0.001	0.63	3.42	1.35	6.0	1.2	0.8	0.2	0.0	0.0	0.0
	Male	15.16	7.04				0.35	0.66	18.1	6.6	1.3	0.3	0.0	0.0	0.0
ALC	Female	1.92	2.49	−10.55	<0.001	0.74	4.49	1.91	3.5	1.5	0.2	0.0	0.0	0.0	0.0
	Male	4.65	4.59				3.82	1.77	13.4	4.5	2.6	1.1	1.0	0.0	0.0
DRG	Female	2.37	3.04	−4.41	<0.001	0.30	8.50	2.21	5.6	1.4	0.4	0.4	0.0	0.2	0.0
	Male	3.41	3.78				6.62	2.02	7.9	2.9	1.0	0.8	0.6	0.3	0.0
Treatment scales															
AGG	Female	13.48	6.95	−3.66	<0.001	0.25	0.63	0.76	8.4	3.6	0.2	0.2	0.0	0.0	0.0
	Male	15.23	7.28				0.04	0.51	15.0	3.7	1.0	0.0	0.0	0.0	0.0
SUI	Female	3.23	5.53	−1.67	0.099	0.11	9.85	2.90	6.2	2.9	1.4	0.6	0.6	0.0	0.0
	Male	3.88	5.97				5.07	2.25	6.6	5.0	2.1	0.6	0.3	0.0	0.0
STR	Female	6.98	3.60	0.24	0.815	0.01	0.81	0.78	11.0	3.5	0.6	0.2	0.0	0.0	0.0
	Male	6.93	3.75				1.36	0.91	11.5	2.6	1.0	0.6	0.0	0.0	0.0
NON	Female	6.59	4.01	−3.94	<0.001	0.27	−0.20	0.51	14.5	3.0	0.4	0.0	0.0	0.0	0.0
	Male	7.65	3.90				−0.47	0.31	19.4	5.0	0.0	0.0	0.0	0.0	0.0
RXR	Female	15.18	4.13	−0.27	0.791	0.02	0.25	−0.62	13.1	0.2	0.0	0.0	0.0	0.0	0.0
	Male	15.26	4.33				−0.06	−0.57	16.8	0.3	0.0	0.0	0.0	0.0	0.0
Interpersonal scales															
DOM	Female	19.91	5.31	−1.82	0.066	0.12	−0.03	−0.06	11.7	2.9	0.0	0.0	0.0	0.0	0.0
	Male	20.54	4.90				−0.14	−0.14	12.3	1.8	0.0	0.0	0.0	0.0	0.0
WRM	Female	21.29	5.62	2.02	0.043	0.14	−0.28	−0.16	15.1	3.1	0.0	0.0	0.0	0.0	0.0
	Male	20.54	5.47				−0.22	−0.03	11.5	2.1	0.0	0.0	0.0	0.0	0.0

Participation was voluntary and all participants read and signed the informed consent form prior to taking part in the study. Participants did not receive compensation for their involvement in the study.

### Measures

Personality Assessment Inventory (PAI; Morey, 1991). The PAI is a self-report personality inventory comprised of 344 items, organized into 22 scales (i.e., 4 validity scales, 11 clinical scales, 5 treatment consideration scales, and 2 interpersonal scales), and 10 subscales (Morey, 1991). The Portuguese version of the PAI was developed and made available by Hogrefe Publishing, which approved the current adaptation and validation study (e.g., translation, back translation, preliminary version, and normative data).

### Statistical analyses

The statistical analyses were performed using the IBM Statistical Package for the Social Sciences Statistics (SPSS) version 26. Group differences were analyzed using Student’s *t* test and analysis of variance (ANOVA, Tukey *post hoc*). Cohen’s *d* and partial *η*^2^ were calculated to determine the effect size of the difference between groups.

To determine the influence of gender, age, and education level on PAI scores, linear regression analyses were also conducted. The total variance (*R^2^*) of the regression model and the standardized (*β*) regression coefficients for each independent variable were calculated.

## Results

### Portuguese normative data and comparison with other international versions

The *M* and *SD* of the PAI raw scores for the Portuguese standardization sample are presented in Table 4. As expected, the raw scores of the clinical scales were low when compared to the maximum scores. As Morey (1991) suggested, these scales measure pathological content, which is expected to be limited in a community-based sample (Morey, 1991).

**Table 4:** Descriptive statistics for the Portuguese standardization sample and for international samples raw scores

Scale	Portuguese sample		U.S. sample				German sample				Greek sample			
Validity	*M*	*SD*	*M*	*SD*	*p*	*d*	*M*	*SD*	*p*	*d*	*M*	*SD*	*p*	*d*
ICN	5.49	2.38	5.49	3.39	1.00	.00	3.45	2.2	<.001	.81	05.50	3.45	.17	.05
INF	3.40	2.06	2.66	2.57	<.001	.36	2.39	2.0	<.001	.49	3.46	2.77	.41	.03.
NIM	2.02	2.50	1.69	2.70	<.001	.13	1.81	2.5	.01	.08	1.39	2.90	<.001	.25
PIM	15.68	3.95	15.07	4.36	<.001	.15	16.72	4.3	<.001	.26	14.86	4.66	<.001	.21
Clinical														
SOM	13.29	9.32	11.09	10.07	<.001	.24	13.44	11.	.64	.02	10.14	10.17	<.001	.34
ANX	23.33	12.16	16.47	10.56	<.001	.56	18.04	10.	<.001	.44	16.97	10.49	<.001	.52
ARD	21.89	9.60	19.91	8.30	<.001	.21	21.02	8.3	.01	.09	20.20	8.50	<.001	.18
DEP	19.65	10.70	14.28	9.43	<.001	.50	17.17	10	<.001	.23	14.53	9.49	<.001	.48
MAN	21.23	8.65	23.01	9.22	<.001	.21	22.16	9.0	<.01	.11	25.11	10.32	<.001	.45
PAR	24.51	9.69	18.45	8.69	<.001	.62	22.26	8.9	<.001	.23	18.25	8.72	<.001	.65
SCZ	15.96	8.29	13.99	7.79	<.001	.24	13.38	7.4	<.001	.31	14.29	7.89	<.001	.20
BOR	23.09	10.39	18.03	18.03	<.001	.49	18.64	9.3	<.001	.43	18.18	9.89	<.001	.47
ANT	12.83	6.67	13.16	9.11	<.001	.05	11.74	8.2	<.001	.16	13.75	9.17	<.001	.14
ALC	3.08	3.79	4.83	5.62	<.001	.46	4.58	5.1	<.001	.40	4.57	5.58	<.001	.39
DRG	2.81	3.41	4.09	4.99	<.001	.37	3.90	4.2	<.001	.32	4.21	5.01	<.001	.41
Treatment														
AGG	14.22	7.14	14.81	8.42	.01	.08	13.37	7.3	<.001	.12	15.86	8.50	<.001	.23
SUI	3.50	5.73	3.28	4.86	.24	.04	3.08	4.8	.03	.07	3.07	4.81	.02	.08
NON	7.04	3.99	5.80	4.45	<.001	.31	6.45	3.9	<.001	.15	5.18	3.32	<.001	.47
STR	6.96	6.96	3.66	4.90	<.001	.56	5.08	4.1	<.001	.51	5.95	4.50	<.001	.28
RXR	15.21	15.21	4.21	13.76	<.001	.34	15.84	4.1	<.001	.15	14.00	4.67	<.001	.29
Interperso														
DOM	20.18	5.15	20.60	5.59	.02	0.8	19.04	5.1	<.001	.22	19.98	5.62	.24	.04
WRM	20.97	5.57	23.48	23.48	<.001	.4	22.09	5.4	<.001	.20	23.60	23.6	<.001	.45

A one-sample t-test was conducted to compare the raw scores of all the 22 PAI scales for the Portuguese version to other international versions. The raw scores of the Portuguese sample were statistically higher than the U.S. raw scores, except for the MAN, ANT, ALC, DRG, AGG, DOM, and WRM scales (U.S. > Portuguese, *p* < 0.05) (see Table 4). Non-significant differences were only found in three scales: ICN, ANT, and SUI. The magnitude of the differences ranged from very small (e.g., *d* = 0.00 in the ICN scale) to medium (e.g., *d = 0*.62 in the PAR scale).

Table 4 also presents comparisons between raw scores of the Portuguese version and other two European versions (Greek and German). Compared to the Portuguese sample, the Greek normative sample scored lower in two validity scales (NIM and PIM). In the clinical scales the Portuguese version revealed statistically higher scores in all scales, except for MAN, ANT, ALC, and DRG (Greek > Portuguese, *p* < 0.001). The same can be found for the treatment scales, where the Portuguese sample scored higher, except for in AGG. Finally, with regards to the interpersonal scales, the Greek sample scored higher in WRM. Effect sizes ranged from *d* = 0.03 to 0.65. In the comparisons between the Portuguese and German versions, we found that the Portuguese sample scored statistically higher in all validity and clinical scales except for PIM, MAN, DRG, and ALC (German > Portuguese, *p* < 0.001). Similarly, the Portuguese sample also scored higher in all treatment and interpersonal scales, except for RXR and WRM. Effect sizes ranged from *d* = 0.02 to 0.81.

### Differences and predictive effect of sociodemographic variables over the PAI scales

The analysis of the relationships between the PAI scales and sociodemographic variables showed that gender, age, and educational level had significant effects in some PAI scales.

As shown in Table 3, gender appears to exhibit some degree of influence on PAI scores. Men scored significantly higher in ICN, INF, MAN, PAR, ANT, ALC, DRG, AGG, and NON, while women scored higher in SOM, ANX, ARD, and WRM. No significant differences were found for the remaining scales. Following methodology employed by Ingram et al. (2020) in their study with the MMPI-2-RF, cumulative elevation frequencies associated with various *T* score cutoffs, across gender, are presented in Table 3.

In relation to age, the youngest age group (i.e., between 18 and 24 years) scored higher on the ICN, ANX, ARD, DEP, MAN, PAR, SCZ, BOR, ANT, DRG, SUI, STR, and NON scales when compared to older age groups In contrast, older age groups (e.g., between 60 and 74 years) scored significantly higher in PIM, AGG, RXR, DOM, and WRM. In the NIM scale mixed findings were found with participants aged 18 to 24 scoring higher when compared to people older than 40 years, but individuals aged 60 to 74 scored higher when compared to people aged 25 to 39 (see Table 5). Effect sizes ranged from small (partial *η*^2^ = 0.002 for SOM) to medium to (partial *η*^2^ = 0.090 for ANX) (Cohen, 1988; Richardson, 2011).

**Table 5:** PAI normative data for the Portuguese sample by age (raw scores).

	18–24 *M*(*SD*)	25–29 *M*(*SD*)	30–39 *M*(*SD*)	40–49 *M*(*SD*)	50–59 *M*(*SD*)	60–74 *M*(*SD*)	*F*	*p*	Partial η^2^	Differences between means (Cohen’s *d*)
Validity scales										
ICN	6.10 (2.58)	5.77 (2.39)	5.23 (2.52)	5.31 (2.19)	5.17 (2.25)	5.20 (2.41)	3.11	0.01	0.017	18–24 > 30–39 (0.34) 18–24 > 50–59 (0.38) 18–24 > 60–74 (0.36)
INF	3.53 (2.15)	3.13 (2.18)	3.47 (2.01)	3.42 (2.12)	3.34 (2.14)	3.44 (1.80)	0.49	0.79	0.003	–
NIM	3.06 (2.98)	2.34 (3.00)	2.21 (2.65)	1.87 (2.33)	1.72 (2.12)	1.37 (1.92)	6.98	<0.001	0.038	18–24 > 40–49 (0.45) 18–24 > 50–59 (0.52) 18–24 > 60–74 (0.68) 25–29 < 60–74 (0.39) 30–39 < 60–74 (0.36)
PIM	14.22 (4.60)	14.25 (3.90)	15.02 (3.82)	15.82 (4.06)	16.47 (3.23)	17.30 (3.38)	13.22	<0.001	0.069	18–24 < 40–49 (0.37) 18–24 < 50–59 (0.57) 18–24 < 60–74 (0.76) 25–29 < 40–49 (0.39) 25–29 < 50–59 (0.62) 25–29 < 60–74 (0.84) 30–39 < 50–59 (0.41) 30–39 < 60–74 (0.66) 40–49 < 60–74 (0.42)
Clinical scales										
SOM	13,74 (9.35)	12.45 (9.49)	13.66 (10.34)	13.41 (9.41)	13.41 (8.63)	12.69 (8.63)	0.37	0.87	0.002	–
ANX	30.48 (14.71)	28.13 (13.27)	24.42 (11.79)	21.91 (12.01)	20.43 (9.51)	19.29 (9.25)	17.64	<0.001	0.090	18–24 > 30–39 (0.45) 18–24 > 40–49 (0.64) 18–24 > 50–59 (0.81) 18–24 > 60–74 (0.91) 25–29 > 40–49 (0.49) 25–29 > 50–59 (0.67) 25–29 > 60–74 (0.77) 30–39 > 50–59 (0.37) 30–39 > 60–74 (0.45)
ARD	26.18 (11.81)	24.70 (9.84)	22.46 (10.09)	20.80 (8.93)	20.22 (8.17)	19.89 (8.12)	9.17	<0.001	0.049	18–24 > 30–39 (0.34) 18–24 > 40–49 (0.51) 18–24 > 50–59 (0.59) 18–24 > 60–74 (0.62) 25–29 > 40–49 (0.41) 25–29 > 50–59 (0.50) 25–29 > 60–74 (0.53)
DEP	22.74 (12.32)	21.44 (12.88)	20.16 (11.14)	18.68 (10.67)	18.57 (8.58)	18.37 (9.12)	3.53	0.01	0.019	18–24 > 40–49 (0.35) 18–24 > 50–59 (0.39) 18–24 > 60–74 (0.40)
MAN	23.39 (9.48)	20.10 (8.06)	21.61 (8.57)	21.25 (8.73)	21.09 (8.88)	19.98 (7.77)	2.32	0.04	0.013	18–24 > 60–74 (0.39)
PAR	26.34 (9.08)	24.25 (10.10)	24.99 (9.75)	25.10 (9.92)	24.49 (9.75)	21.76 (9.00)	3.33	0.01	0.018	18–24 > 60–74 (0.51) 30–39 > 60–74 (0.34) 40–49 > 60–74 (0.35)
SCZ	20.21 (10.02)	16.24 (9.41)	16.87 (8.43)	15.28 (8.29)	14.92 (6.86)	13.76 (6.07)	9.34	<0.001	0.050	18–24 > 25–29 (0.41) 18–24 > 30–39 (0.36) 18–24 > 40–49 (0.54) 18–24 > 50–59 (0.62) 18–24 > 60–74 (0.78) 30–39 > 60–74 (0.42)
BOR	27.69 (12.19)	26.14 (11.41)	23.75 (10.20)	22.40 (10.18)	21.64 (8.54)	19.66 (9.12)	10.32	<0.001	0.055	18–24 > 30–39 (0.35) 18–24 > 40–49 (0.47) 18–24 > 50–59 (0.57) 18–24 > 60–74 (0.75) 25–29 > 40–49 (0.35) 25–29 > 50–59 (0.45) 25–29 > 60–74 (0.63) 30–39 > 60–74 (0.42)
ANT	15.28 (7.58)	12.36 (6.00)	13.01 (6.20)	12.93 (7.36)	12.63 (6.50)	11.09 (5.38)	5.07	<0.001	0.028	18–24 > 25–29 (0.43) 18–24 > 40–49 (0.31) 18–24 > 50–59 (0.38) 18–24 > 60–74 (0.64)
ALC	2.44 (2.90)	2.99 (2.98)	3.20 (3.70)	3.27 (4.20)	3.07 (3.63)	3.16 (4.42)	0.78	0.57	0.004	–
DRG	3.75 (4.47)	3.06 (3.63)	3.03 (3.23)	2.45 (3.38)	2.67 (3.00)	2.41 (2.93)	2.84	0.05	0.016	18–24 > 40–49 (0.33) 18–24 > 60–74 (0.35)
Treatment scales										
AGG	13.88 (7.44)	11.89 (6.89)	15.22 (7.43)	14.56 (7.05)	14.68 (6.90)	13.64 (6.85)	3.07	0.01	0.017	25–29 < 30–39 (0.46) 25–29 < 40–49 (0.38) 25–29 < 50–59 (0.40)
SUI	5.31 (6.81)	3.89 (6.20)	3.86 (6.37)	3.24 (5.70)	2.80 (4.42)	2.71 (4.83)	3.55	0.01	0.019	18–24 > 40–49 (0.33) 18–24 > 50–59 (0.44) 18–24 > 60–74 (0.44)
STR	7.55 (3.36)	7.36 (3.55)	6.98 (3.55)	7.05 (3.66)	7.18 (3.86)	5.82 (3.66)	3.68	<0.01	0.020	18–24 > 60–74 (0.49) 25–29 > 60–74 (0.43) 40–49 > 60–74 (0.34) 50–59 > 60–74 (0.36)
NON	7.97 (4.19)	5.98 (3.56)	6.80 (4.16)	7.14 (4.15)	7.10 (3.98)	7.05 (3.51)	2.61	0.02	0.014	18–24 > 25–29 (0.51)
RXR	13.36 (5.15)	13.78 (4.55)	14.84 (3.96)	15.41 (3.90)	15.96 (3.93)	16.78 (3.59)	12.24	<0.001	0.064	18–24 < 30–39 (0.32) 18–24 < 40–49 (0.45) 18–24 < 50–59 (0.57) 18–24 < 60–74 (0.77) 25–29 < 40–49 (0.38) 25–29 < 50–59 (0.52) 25–29 < 60–74 (0.73) 30–39 < 60–74 (0.51) 40–49 < 60–74 (0.37)
Interpersonal scales										
DOM	18.58 (6.33)	18.56 (5.18)	19.77 (5.27)	20.32 (4.84)	21.26 (4.70)	21.39 (4.30)	7.40	<0.001	0.040	18–24 < 40–49 (0.31) 18–24 < 50–59 (0.48) 18–24 < 60–74 (0.52) 25–29 < 50–59 (0.55) 25–29 < 60–74 (0.59)
WRM	19.36 (5.56)	20.16 (6.23)	19.94 (5.45)	21.58 (5.66)	21.94 (5.46)	21.87 (4.75)	5.82	<0.001	0.032	18–24 < 40–49 (0.40) 18–24 < 50–59 (0.47) 18–24 < 60–74 (0.49) 30–39 < 40–49 (0.30) 30–39 < 50–59 (0.37) 30–39 < 60–74 (0.38)

Education level also appears to have an effect across scales, but mostly for participants who attended 10th to 12th grades. As seen in Table 6, this group scored significantly higher in ICN, NIM, SOM, ANX, ARD, DEP, PAR, SCZ, BOR, ANT, AGG, SUI, STR and NON, when compared to participants who attended University. On the other hand, individuals who attended 7th to 9th grade scored higher than people who attended University in INF, ARD, PAR, and SCZ. Participants who attended University only registered higher scores in RXR and DOM when compared with participants that attended 10th to 12th grades. Effect sizes ranged from small (partial *η*^2^ = 0.001 for MAN) to medium (partial *η*^2^ = 0.057 for PAR) (Cohen, 1988; Richardson, 2011).

**Table 6:** PAI normative data for the Portuguese sample by educational level (raw scores).

	1st to 6th grade M(SD)	7th to 9th grade M(SD)	10th to 12th grade M(SD)	University M(SD)	*F*	*p*	Partial *η*^2^	Differences between means (Cohen’s *d*)
Validity scales								
ICN	5.41 (2.46)	5.60 (2.44)	5.72 (2.37)	5.16 (2.36)	3.75	0.01	0.012	10–12 > U (0.24)
INF	3.68 (1.99)	4.04 (2.37)	3.41 (2.12)	3.30 (1.98)	2.77	0.04	0.009	7–9 > U (0.34)
NIM	1.86 (2.62)	2.25 (2.76)	2.54 (2.90)	1.68 (2.11)	7.98	<0.001	0.026	10–12 > U (0.34)
PIM	16.82 (3.39)	16.06 (4.31)	15.20 (3.99)	15.87 (3.87)	2.71	0.04	0.009	–
Clinical scales								
SOM	15.14 (8.75)	14.37 (9.43)	15.08 (9.23)	11.98 (8.75)	7.87	<0.001	0.026	10–12 > U (0.33)
ANX	22.36 (8.74)	23.66 (11.58)	25.78 (13.56)	21.84 (11.22)	6.87	<0.001	0.022	10–12 > U (0.32)
ARD	23.18 (8.78)	23.96 (9.44)	23.37 (10.10)	20.65 (9.14)	6.54	<0.001	0.021	7–9 > U (0.36) 10–12 > U (0.28)
DEP	20.77 (7.89)	20.54 (10.46)	22.42 (12.23)	17.80 (9.40)	12.63	<0.001	0.041	10–12 > U (0.42)
MAN	19.68 (7.42)	21.56 (9.21)	21.14 (8.19)	21.31 (8.91)	0.29	0.83	0.001	–
PAR	27.05 (9.57)	27.25 (10.25)	27.09 (9.08)	22.45 (9.52)	18.12	<0.001	0.057	7–9 > U (0.49) 10–12 > U (0.50)
SCZ	17.14 (6.22)	19.07 (10.13)	17.80 (8.55)	14.37 (7.57)	15.26	<0.001	0.049	7–9 > U (0.53) 10–12 > U (0.43)
BOR	23.18 (10.87)	24.01 (11.19)	25.36 (10.51)	21.57 (9.93)	8.89	<0.001	0.029	10–12 > U (0.24)
ANT	12.77 (4.89)	13.66 (7.29)	13.95 (7.27)	12.03 (6.16)	5.75	<0.01	0.019	10–12 > U (0.29)
ALC	3.27 (4.26)	2.79 (2.68)	3.37 (4.15)	2.92 (3.65)	1.04	0.39	0.003	–
DRG	1.82 (2.70)	3.72 (4.18)	3.04 (3.75)	2.60 (3.07)	3.38	0.02	0.011	–
Treatment scales								
AGG	12.82 (7.15)	14.43 (6.62)	15.55 (7.44)	13.44 (6.91)	5.96	<0.01	0.020	10–12 > U (0.29)
SUI	2.32 (2.95)	3.56 (5.55)	4.50 (6.54)	2.94 (5.22)	5.11	<0.01	0.017	10–12 > U (0.26)
STR	6.82 (3.23)	7.18 (3.38)	7.88 (3.74)	6.38 (3.56)	11.18	<0.001	0.036	10–12 > U (0.41)
NON	7.18 (4.15)	7.75 (3.92)	7.75 (4.07)	6.50 (3.88)	7.07	<0.001	0.023	10–12 > U (0.31)
RXR	16.73 (3.49)	15.72 (4.36)	14.53 (4.47)	15.49 (4.01)	4.73	<0.01	0.016	10–12 < U (0.22)
Interpersonal scales								
DOM	18.27 (3.71)	19.91 (5.23)	19.36 (5.21)	20.80 (5.07)	6.15	<0.001	0.020	10–12 < U (0.28)
WRM	21.14 (5.51)	19.79 (6.40)	20.46 (5.44)	21.44 (5.51)	3.08	0.03	0.010	

1–4 = 1st to 4th grade; 5–6 = 5th to 6th grade; 10–12 = 10th to 12th grade; U = University.

Linear regression analyses were also conducted to analyze the predictive effect of gender, age, and education level on PAI scores. The beta weights, presented in Table 7, suggested that gender contributes most to predicting scores in INF, MAN, ANT, ALC, DRG, AGG, and NON. Age was the most relevant predictor in ICN, NIM, PIM, ANX, ARD, SCZ, BOR, SUI, RXR, DOM, and WRM. On the other hand, education level contributes most in predicting scores in SOM, DEP, PAR, and STR. The highest adjusted mean *R^2^* value is 0.13 for ALC, which indicates that 13% of the variance on the ALC scores was explained by these three predictors.

**Table 7:** Linear regression analysis summary for gender, age, and education predicting PAI scores.

	Variable	*β*	*R^2^*	*p*
Validity scales				
ICN	Gender	0.097**	0.03	<0.001
	Age	−0.112**		
	Education	−0.079*		
INF	Gender	0.089**	0.01	0.01
	Age	−0.004		
	Education	−0.075*		
NIM	Gender	−0.032	0.05	<0.001
	Age	−0.189***		
	Education	−0.113**		
PIM	Gender	0.017	0.07	<0.001
	Age	0.258***		
	Education	0.010		
Clinical scales				
SOM	Gender	−0.108**	0.03	<0.001
	Age	−0.014		
	Education	−0.138***		
ANX	Gender	−0.159***	0.12	<0.001
	Age	−0.290***		
	Education	−0.102**		
ARD	Gender	−0.148***	08	<0.001
	Age	−0.208***		
	Education	−0.139***		
DEP	Gender	−0.069*	0.04	<0.001
	Age	−0.130***		
	Education	−0.152***		
MAN	Gender	0.090**	0.01	0.01
	Age	−0.080*		
	Education	0.019		
PAR	Gender	0.075*	0.06	<0.001
	Age	−0.107**		
	Education	−0.204***		
SCZ	Gender	−0.008	0.08	<0.001
	Age	−0.207***		
	Education	−0.196***		
BOR	Gender	−0.029	0.07	<0.001
	Age	−0.231***		
	Education	−0.124***		
ANT	Gender	0.299***	0.12	<0.001
	Age	−0.144***		
	Education	−0.088**		
ALC	Gender	0.356***	0.13	<0.001
	Age	0.034		
	Education	−0.007		
DRG	Gender	0.151***	0.04	<0.001
	Age	−0.113**		
	Education	−0.049		
Treatment scales				
AGG	Gender	0.117***	0.02	<0.001
	Age	0.023		
	Education	−0.066*		
SUI	Gender	0.056	0.02	<0.001
	Age	−0.134***		
	Education	−0.060		
STR	Gender	−0.012	0.03	<0.001
	Age	−0.107**		
	Education	−0.126***		
NON	Gender	0.124***	0.03	<0.001
	Age	−0.015		
	Education	−0.116***		
RXR	Gender	0.004	0.06	<0.001
	Age	0.252***		
	Education	0.017		
Interpersonal scales				
DOM	Gender	0.063	0.06	<0.001
	Age	0.194***		
	Education	0.126***		
WRM	Gender	−0.067*	0.04	<0.001
	Age	0.165***		
	Education	0.081*		

*p < 0.05; **p < 0.01; ***p < 0.001.

### Reliability

Table 8 shows the internal consistency (Cronbach alpha) for the PAI scales of the Portuguese standardization sample and other international versions. Overall, the internal consistency of the Portuguese standardization sample revealed lower Cronbach alphas on the validity scales (0.08–0.68), but adequate on the clinical (0.73–0.92, except for DRG), treatment (0.75–0.91, except for STR), and interpersonal (0.78–0.82) scales. We found lower alphas on the DRG and STR scales (0.58 and 0.63, respectively), and in the four validity scales.

**Table 8:** Conbrach’s alpha for the Portuguese and other international versions of the PAI.

	Cronbach’s alpha (*α*)							
	Portuguese sample	U.S. sample	German sample	Spanish sample	South-Korean sample	Chilean sample	Greek sample	Vietnamese sample
Validity scales								
ICN	0.08	0.45	0.73	–	0.68	–	–	0.43
INF	0.19	0.52	0.26	–	0.44	–	–	0.41
NIM	0.67	0.72	0.68	0.60	0.70	–	0.85	0.72
PIM	0.68	0.71	0.71	0.67	0.76	–	0.86	0.80
Clinical scales								
SOM	0.87	0.89	0.91	0.87	0.83	0.86	0.79	0.88
ANX	0.92	0.90	0.89	0.89	0.87	0.85	0.82	0.92
ARD	0.83	0.76	0.74	0.82	0.77	0.79	0.90	0.82
DEP	0.89	0.87	0.88	0.86	0.88	0.85	0.80	0.88
MAN	0.82	0.82	0.81	0.79	0.79	0.79	0.95	0.85
PAR	0.87	0.85	0.83	0.84	0.77	0.78	0.84	0.83
SCZ	0.84	0.81	0.80	0.79	0.79	0.79	0.82	0.84
BOR	0.87	0.87	0.84	0.84	0.85	0.84	0.74	0.87
ANT	0.73	0.84	0.82	0.76	0.75	0.77	0.85	0.84
ALC	0.75	0.84	0.82	0.72	0.81	0.83	0.87	0.64
DRG	0.58	0.74	0.63	0.64	0.60	0.72	0.80	0.68
Treatment scales								
AGG	0.82	0.85	0.81	0.83	0.75	0.80	0.83	0.85
SUI	0.91	0.85	0.87	0.81	0.80	0.84	0.92	0.94
STR	0.63	0.72	0.74	0.67	0.76	0.66	0.74	0.73
NON	0.75	0.76	0.72	0.66	0.67	0.68	0.86	0.73
RXR	0.77	0.76	0.70	0.75	0.60	0.72	0.84	0.72
Interpersonal scales								
DOM	0.78	0.78	0.76	0.68	0.70	0.70	0.83	0.81
WRM	0.82	0.79	0.78	0.73	0.78	0.67	0.83	0.74

The Portuguese PAI’s internal consistency was also compared to other international versions, namely the U.S., German, Spanish, South-Korean, Chilean, Greek, and Vietnamese versions. Overall, the results showed that the Portuguese version of the PAI showed higher Cronbach alphas than the other international versions in most clinical scales (i.e., ANX, ARD, DEP, MAN, PAR, SCZ, BOR, ANT, ALC, and DRG), except when compared to the Vietnamese version, which revealed similar alphas for ANX, SCZ, and BOR. Internal consistency in the validity scales was somewhat lower than in other countries, particularly when compared to the Greek sample. Regarding the interpersonal scales, the Portuguese version had the second highest Cronbach alpha in WRM, and the third highest in DOM.

## Discussion and conclusions

Psychological test translation and adaptation is a growing phenomenon in the last couple of decades, and relates with several areas of study, including the study of personality. The MMPI (also adapted for the Portuguese population) is the most used measure worldwide (Friedman et al., 2014). Nevertheless, it is worth recognizing that the PAI has been gaining popularity since its birth as a useful, valid, reliable, and robust measure of personality, with multiple benefits and with great potential in several contexts (e.g., clinical, forensic). The PAI has been increasingly referenced in empirical studies centered around the use of personality measures as the second most used inventory in forensic settings (e.g., Ackerman et al., 2021), in the neuropsychology context (e.g., Martin et al., 2015), in the study of malingering (e.g., Schroeder et al., 2016), and in the training of recently graduated psychologists (e.g., Stedman et al., 2017). In the clinical context, it is also widely recognized as the third most used measure of personality (after MMPI-2/MMPI-2-RF and MCMI-III) (e.g., Wright et al., 2017).

PAI’s adaptation and validation to the Portuguese population is of great interest, not only because it would allow further research focused on the PAI, supporting its growth and dissemination, but also because it would allow Portuguese psychologists to use a valid and robust alternative to the MMPI-2-RF, that benefits from adequate reliability (i.e., internal consistency and retest values) and validity (i.e., discriminant and convergent). Compared to measures such as the MMPI-2 and MCMI-III, the PAI use of non-overlapped scales provides distinct advantages, particularly with respect of discriminant validity (Wise et al., 2010). Additionally, in forensic settings its use is particularly valuable due to its ability to correctly predict inmate violence and institutional misconduct (e.g., Reidy et al., 2016).

Comparing the raw scores of the Portuguese and U.S. versions of the PAI, we verified that, in general, the Portuguese sample manifested significantly higher scores in all scales, except in the MAN, ALC, DRG, AGG, DOM, and WRM scales. Furthermore, the largest effect sizes were found in the PAR, ANX, and DEP scales. The Portuguese sample also had higher scores in all clinical samples than the German (Groves and Engel, 2007) and Greek (Lyrakos, 2011) standardization samples, except for SOM, ALC, MAN, ANT, and DRG. These higher scores in the clinical scales may be due to nuances of translation, cultural differences related to the concepts measured by the scales, sampling issues related to the use of an online sample for normative data collection, and/or due to the higher prevalence of mental health disorders found in Portugal. Indeed, Portugal is the second European country with the highest psychiatric disorder prevalence, and anxiety disorders are particularly common (Pinto-Meza et al., 2012; Antunes et al., 2018).

Regarding to gender, women appeared to score significantly higher than men in SOM, ANX, ARD, and WRM, and men scored higher in ICN, INF, MAN, PAR, ANT, ALC, DRG, AGG, and NON. These results are in accordance with literature that associates a higher prevalence of somatization problems (e.g., Wool and Barsky, 1994) and anxiety (e.g., McLean et al., 2011) in women; but manic episodes (e.g., Azorin et al., 2013), antisociality (e.g., Glenn et al., 2013), alcoholism (e.g., Ceylan-Isik et al., 2010), and drug abuse (e.g., Cotto et al., 2010) in men. Furthermore, our results are in line with the original manual of the PAI, where Morey (1991) also found higher scores in the SOM, ANX, ARD, and WRM scales in women, but lower in ICN, INF, MAN, PAR, ANT, ALC, DRG, AGG, and NON.

Differences in age showed that younger participants appeared to score higher in scales such as ICN, NIM, ANX, ARD, DEP, MAN, PAR, SCZ, BOR, ANT, DRG, SUI, STR, and NON, and that older participants tended to score higher in PIM, AGG, RXR, DOM, and WRM. These data were consistent with Morey’s (2007) findings that younger adults tend to score higher in PAR, BOR and ANT, and also with other studies that attributes less personality stability in younger ages and that recognizes a greater prevalence of personality disorders in these age groups (e.g., Johnson et al., 2000).

The effect of education level on PAI scores was unexpected. Contrary to what is reported in the original manual, in our study, the groups with lower years of education (i.e., 1st to 6th grade) did not score higher than the groups with higher levels of education (Morey, 1991). Instead, our results revealed significant differences between respondents who completed 7th to 9th grades and individuals who attended University (7th to 9th grades scored higher in INF, ANX, PAR, and SCZ), and between participants who completed 10th to 12th and those who attended University (10th to 12th grades scored higher in ICN, NIM, SOM, ANX, ARD, DEP, PAR, SCZ, BOR, ANT, AGG, SUI, STR, and NON; but participants who attended University scored higher in RXR and WRM). Therefore, although these results generally reproduce the expected effect (i.e., lower education level leading to higher scores), they do not reproduce it in the way it was expected (i.e., participants who completed 1st to 6th grade did not score significantly higher than people who attended University), which could reflect differences in expectations around education in the two cultures.

The present study also aimed to investigate the psychometric properties of the PAI to the Portuguese population by analyzing its reliability. Furthermore, similarly to what other studies with international versions of the PAI did (e.g., Jeffay et al., 2021, for the French-Canadian version), we compared the newly developed Portuguese version with the original U.S. sample, as well as with other international adaptations of the inventory. Our sample (*n* = 900), albeit smaller than some international samples (e.g., Chinese, *n* = 1884; Italian, *n* = 1,548; French-Canadian, *n* = 1,120), is similar to the Argentinian (*n* = 998) and Mexican (*n* = 961) samples, and higher than other international samples (e.g., German, *n* = 749; Chilean, *n* = 569; Iranian, *n* = 462).

Regarding to the Portuguese PAI’s reliability, the results indicated overall adequate internal consistency. The Portuguese version revealed higher Cronbach alphas than the U.S. version in 9 out of 22 scales of the PAI, but lower values in the remaining 13 scales (Cronbach alphas <0.70 were identified in 7 scales: ICN, INF, NIM, PIM, DRG, and STR). Some Cronbach alphas were very low, particularly ICN, INF, and DRG; however, most of these scales are validity scales, which is consistent with the assertion that the ICN and INF scales possibly measuring variance error indicators and, therefore, leading to low internal consistency values (Morey, 1991). The lower Cronbach alphas observed in the NIM, PIM, and DRG scales were similar, albeit smaller, than the values found in the German version (Groves and Engel, 2007), and the DRG alpha was similar to the South-Korean (Lee et al., 2020) and higher than the French-Canadian alphas (Jeffay et al., 2021). The smaller STR values were also similar to the alphas reported in the Chilean version (Ortiz-Tallo et al., 2011).

The reliability data also suggested that the Portuguese version showed higher alphas in most clinical scales (except for SOM, ARD, MAN, ANT, ALC, and DRG), when compared to the other versions. The Vietnamese version, however, presented the same values in ANX, SCZ, and BOR. The highest internal consistency values in the Portuguese version of the PAI (i.e., > 0.85) belong to the SOM, ANX, DEP, PAR, BOR, and SUI scales, and these alphas were overall higher than in other international versions. In general, the scales of the Portuguese demonstrated internal consistency of the same or greater magnitude than the other international versions of the PAI.

Despite the strengths of the present study, it is not without some limitations. The sample was collected entirely online, which may be problematic. Indeed, online data collection has been a growing phenomenon in the last decade, because of an increase in demands for higher statistical power associated with larger sample sizes (Sassenberg and Ditrich, 2019). Online experiments have several advantages by reducing costs, providing procedure uniformity, and allowing wider accessibility, while also maintaining ethical standards (Dandurand et al., 2008). So, although it may bring some disadvantages (e.g., increased environmental variability during testing, possible multiple submissions, and bigger drop-out rates), many of these can be easily avoidable (e.g., by asking participants to participate in the study in a particular environment, by asking for personal data, and by using IP address verification). In addition, some studies have shown that self-selected web samples do not differ systematically from samples obtained from other ways (e.g., in-person), even for cognitive and perceptual demanding tasks (Dandurand et al., 2008; Germine et al., 2012; Hilbig, 2016; Corey and Ben-Porath, 2020; Agarwal et al., 2023), although other studies using the PAI suggest that online participants may be somewhat more socially withdrawn than those participating in in-person studies (McCredie and Morey, 2019). Future studies should compare results between the data collected online and in-person, to check for possible differences in the normative data and psychometric properties. The analysis of normative data on supplemental indexes (e.g., MFI, CBS) would also contribute to validation efforts of these response style measures and reinforce their utility in many settings. An analysis of creating norms based on sociodemographic variables such as gender, age, and educational level may be equally relevant. Differences in scores among minority groups should be further examined in future studies, with regards to clinical and validity scales. Furthermore, it would be also interesting to include forensic samples (e.g., victims, offenders, forensic professionals) and clinical samples, considering that they may influence the psychometric properties and the normative data of the PAI.

In conclusion, this study provides evidence regarding the psychometric properties of the Portuguese version of the PAI. It is a crucial step into the Portuguese adaptation and validation of this instrument, a measure with considerable potential in clinical, forensic, and research contexts. This adaptation may lead to the growth and development of the psychological assessment field in Portugal, and the opportunity to develop future cross-cultural studies with other international versions of the PAI.

## Data availability statement

The supporting data presented in this article is not readily available because of ethical and commercial rights (Hogrefe Publisher, Portugal). Requests to access the datasets should be directed to Hogrefe Publisher (Portugal).

## Ethics statement

The studies involving humans were approved by Faculty of Psychology and Education Sciences of the University of Coimbra Ethics Board (CEDI/FPCEUC:78/R_5). The studies were conducted in accordance with the local legislation and institutional requirements. The participants provided their written informed consent to participate in this study.

## Author contributions

MP: Writing – original draft, Writing – review & editing. MM: Writing – original draft, Writing – review & editing. OM: Investigation, Methodology, Supervision, Writing – review & editing. DR: Conceptualization, Writing – review & editing. LM: Conceptualization, Formal analysis, Supervision, Validation, Writing – review & editing. MS: Conceptualization, Formal analysis, Investigation, Supervision, Validation, Writing – review & editing, Writing – original draft.

## Footnotes

^1^For example, according to the Portuguese Census, in 2021, 47.6% of the Portuguese population was male, and 52.4% was female.
